# Epidemic of *Klebsiella pneumoniae* ST11 Clone Coproducing KPC-2 and 16S rRNA Methylase RmtB in a Chinese University Hospital

**DOI:** 10.1186/1471-2334-12-373

**Published:** 2012-12-23

**Authors:** Jun-Jie Li, Zi-Ke Sheng, Mei Deng, Sheng Bi, Fei-Shu Hu, Hai-Feng Miao, Zhong-Kang Ji, Ji-Fang Sheng, Lan-Juan Li

**Affiliations:** 1 State Key Laboratory for Diagnosis and Treatment of Infectious Disease, First Affiliated Hospital, College of Medicine, Zhejiang University, Hangzhou, Zhejiang, 310003, People’s Republic of China; 2 Institute of Antibiotics, Huashan Hospital, Fudan University, Shanghai, People’s Republic of China

**Keywords:** Carbapenem, Aminoglycoside, KPC, *rmtB*, Epidemic

## Abstract

**Background:**

Emergence of *rmtB*-positive *Klebsiella pneumoniae* carbapenemase (KPC)-producing *K. pneumoniae* (KPC-KP) poses a great threat to antimicrobial treatment options.

**Methods:**

From January 2010 to December 2010, non-duplicate KPC-KP isolates from our hospital were screened for *rmtB* and multiple other resistance determinants with PCR. Subsequent studies included MIC determination, PFGE, and multilocus sequence typing. Records from patients with KPC-KP isolated were retrospectively reviewed. Comparisons of molecular and clinical characteristics between *rmtB-*positive and *rmtB*–negative isolates were systematically performed, as well as the environmental colonization study in ICU wards.

**Results:**

A total of 84 KPC-KP strains were collected, including 48 *rmtB*-positive KPC-KP (RPKP) and 36 *rmtB*-negative KPC-KP (RNKP) isolates. All KPC-KP isolates were multidrug resistant, with colistin and tigecycline being the most active agents. Compared with RNKP, RPKP displayed a much severer resistance phenotype. Susceptibility rates for amikacin (0% for RPKP versus 88.9% for RNKP, *p* < 0.01), fosfomycin (8.5% for RPKP versus 88.9% for RNKP, *p* < 0.01), and minocycline (6.7% for RPKP versus 52.8% for RNKP, *p* < 0.01), were all significantly lower in RPKP strains. Isolates belonging to PFGE pulsetype A and sequence type 11 were predominant in both groups, including 39 (81.3%) RPKP and 22 (61.1%) RNKP isolates. Nevertheless, RNKP showed more complex genetic backgrounds compared with RPKP. Diverse clinical characteristics were found in both cohorts, however, no significant differences were observed between RPKP and RNKP patients.

**Conclusions:**

RPKP strains have spread widely and gradually replaced RNKP in our hospital. They seemed to show much severer resistance phenotypes compared with RNKP and had a bigger dissemination potential. Prudent use of available active agents combined with good control practices is therefore mandatory.

## Background

During the past 10 years, Chinese clinicians have witnessed a dramatic increase in the rates of carbapenem resistance among clinical isolates of *Klebsiella pneumoniae*. According to Mohnarin and CHINET, two leading antimicrobial resistance surveillance networks in China that cover all regions except Tibet, carbapenem resistance rates among *K. pneumoniae* escalated from 0.7% in 2000 to 2.9% in 2009 [1]. Moreover, in a university hospital in Shanghai, the incidence of carbapenem-resistant *K. pneumoniae* in 2009 reached 12.9% [2]. In China, carbapenem resistance in *K. pneumoniae* has mainly resulted from the rapid dissemination of *Klebsiella pneumoniae* carbapenemase (KPC) [2-4], and ST11 was demonstrated to be the dominant clone of KPC-producing *K. pneumoniae* (KPC-KP) [4].

KPC-KP isolates were usually multidrug resistant, but might remain susceptible to one or more aminoglycoside agents as well as to colistin and/or tigecycline [4-6]. Nevertheless, 16S rRNA methylases (ArmA and RmtB) were reported in a few KPC-producing *Enterobacteriaceae* recently, conferring high level resistance to almost all clinically important aminoglycosides [7-9]. Co-production of 16S rRNA methylases in KPC-producing pathogens and their potential spread could leave few choices for antimicrobial treatment.

Since its first isolation in 2007 [10], KPC-KP has been increasingly emerging at our hospital. Most of these isolates were still susceptible to one or more aminoglycoside antibiotics until 2009; nevertheless, high-level aminoglycoside resistance has emerged and gradually become prevalent in the last two years (data not shown). In May 2010, an *Enterobacter amnigenus* as well as a *K. pneumoniae* isolate, both co-producing *bla*_KPC_ and *rmtB* on a single plasmid, were simultaneously identified from a patient in our neurosurgery ward. And a small outbreak of KPC-RmtB-producing *K. pneumoniae* was revealed in the neurosurgery department [7]. This promoted us to perform a hospital-wide screening of *rmtB* gene in KPC-KP strains. Our study focused on the comparisons of microbiological, molecular and clinical characteristics between *rmtB*-positive KPC-KP (RPKP) and *rmtB*-negative KPC-KP (RNKP) isolates.

## Methods

### Bacterial isolates and patients

Between January 2010 and December 2010, all clinical *K. pneumoniae* isolates exhibiting non-susceptibility to carbapenems (inhibition diameter <16 mm for at least one of meropenem and imipenem with the help of disc diffusion method) were obtained in the First Affiliated Hospital, School of Medicine, Zhejiang University. This hospital is a 2500-bed tertiary-care academic medical center receiving about 2 560 000 outpatients and 71 000 inpatients annually. Detection of *bla*_KPC_ and *rmtB* genes was conducted by PCR and subsequent sequencing based on established methods [4,11]. A case patient was defined as patient with KPC-KP (including RPKP and RNKP) isolated from clinical specimens during our study period. If more than one isolate were obtained from a patient, only the initial isolate was submitted.

### Cohort study

A retrospective observational cohort study was conducted between RPKP and RNKP patients. Detailed clinical information of case patients was extracted from their medical records and follow-up was possible until discharge from our hospital or death. Isolates identified during the first 72 h after admission were characterized as imported, whereas those identified >72 h after admission were determined as nosocomial transmission during the current hospitalization. Infection was defined according to the criteria from Centers for Disease Control and Prevention (CDC)/National Healthcare Safety Network [12]. Patients without infection but with KPC-KP isolated were considered as colonized. The study was approved by the Institutional Review Board of our hospital.

### Bacterial identification and susceptibility testing

Species identification was performed using the Vitek 2 system (bioMerieux, France). MICs of various antimicrobials were determined by agar dilution method and results were interpreted according to the criteria recommended by Clinical and Laboratory Standards Institute (CLSI) 2010 [13]. With regard to tigecycline, breakpoint for *Enterobacteriaceae* based on FDA was used (MIC ≤2 μg/mL as susceptible). And concentration of 4 μg/mL was used as the breakpoint of resistance for colistin [14].

### Molecular typing

Pulse field gel electrophoresis (PFGE) was performed using *XbaΙ* restriction enzyme on all clinical isolates and PFGE profiles were interpreted by the criteria proposed by Tenover [15]. Multilocus sequence typing (MLST) was carried out according to protocols on the MLST website for *K. pneumoniae* (http://www.pasteur.fr/recherche/genopole/PF8/mlst/Kpneumoniae.html).

### PCR testing and DNA sequencing

PCR analysis was conducted to detect a variety of resistance determinants as described previously, including *bla*_OXA_, *bla*_TEM_, *bla*_SHV_, and *bla*_CTX_ genes [4,16], six more 16S rRNA methylase-encoding genes (*armA*, *rmtA*, *rmtC*, *rmtD*, *rmtE* and *npmA*) [11], and plasmid-mediated quinolone resistance genes (*qnrA*, *qnrB*, *qnrS* and *aac(6’)-Ib-cr*) [16]. The PCR products were subsequently sequenced by dideoxynucleotide chain-termination method by ABI 377 (ABI, U.S.A.) and sequences were compared with the nucleotide sequences from GenBank (http://www.ncbi.nlm.nih.gov/blast/).

### Environmental colonization study

A point prevalence survey of environmental colonization was conducted in the general ICU wards in July 2010 to probe the potential environmental reservoirs of RPKP. Environmental surfaces including medical equipments in the immediate vicinity of patients, communal areas, and contaminated hands of medical staff on shift, were under screening. Swab samples were obtained by repeatedly rubbing designated sites with premoistened swabs. The swabs were then placed in brain and heart infusion (BHI) containing meropenem (1 μg/mL) and incubated overnight at 37°C. Isolated strains were subjected to further analysis as clinical isolates as described above.

### Statistical analysis

Comparative analyses were performed using the χ2 test or the Fischer’s exact test for categoric variables, and Student’s t test for the continuous variables, as appropriate. A 2-tailed P value of <0.05 was considered to indicate statistical significance. Analyses were done with SPSS 17.0 (SPSS Inc, Chicago, USA).

## Results

A total of 84 KPC-KP isolates were identified during the study period. Among them, 48 were confirmed to be RPKP, and the rest RNKP. Medical records were available for 35 RPKP patients and 29 RNKP patients. Comparisons of various clinical characteristics between RPKP and RNKP patients are presented in Table 1. Most patients were severely ill and half were from ICU departments. Respiratory tract was the most common site of infection in both cohorts. Although a majority of KPC-KP isolates were acquired at our hospital, 7 RNKP (24.1%) and 4 RPKP isolates (11.4%) were imported. Another notably feature was that bed transfers were rather common among these patients (2.54 times for RPKP versus 2.52 times for RNKP), even after isolation of KPC-KP (1.57 times for RPKP versus 1.66 times for RNKP). Crude mortality was 22.9% among RPKP patients and 27.6% among RNKP patients, and attributable mortality rates were 19.4% and 17.9%, respectively.

**Table 1:** Univariate analysis for clinical characteristics of patients infected or colonized with RPKP and RNKP isolates

***Characteristics***	***Patients with RPKP (n = 35)***	***Patients with RNKP (n = 29)***	***P value***
Male sex	28(80.0)	21(72.4)	0.79
Age, mean years ± SD	63 ± 13.8	63 ± 18.9	0.988
Ward			
ICU	17(48.6)	17(58.6)	0.68
Medicine	6(17.1)	2(6.9)	0.45
Surgery	12(34.3)	10(34.5)	>0.99
Transferred from another hospital	15(42.9)	13(44.8)	0.92
Total length of stay in any hospital before isolation of KPC-KP,			
mean days ± SD	18.7 ± 15.1	16.2 ± 15.0	0.525
Total length of stay until discharge or death, mean days ± SD	36.1 ± 22.2	35.6 ± 22.4	0.94
Route of acquisition of KPC-KP			0.203
Imported	4(11.4)	7(24.1)	--
Acquired in our hospital	31(88.6)	22(75.9)	--
Site of KPC-KP isolation			
Bronchial secretion	15(42.9)	12(41.4)	>0.99
Blood	6(17.1)	10(34.5)	0.27
Drainage	7(20.0)	6(20.7)	>0.99
Urine	4(11.4)	1(3.4)	0.38
Cerebrospinal fluid	2(5.7)	0	--
Pus	1(2.9)	0	--
Infection	31(88.6)	28(96.6)	0.37
Median APACHEII score	17(2–37)	16(2–35)	0.129
Median Charlson score	2(0–10)	2(0–5)	0.233
Invasive procedures			
Surgery within a month	16(45.7)	15(51.7)	0.83
Mechanical ventilation	18(51.4)	18(62.1)	0.68
Renal replacement therapy	11(31.4)	9(31.0)	>0.99
Drainage catheters	33(94.3)	20(68.9)	0.46
Total times of bed transfer during this hospitalization, mean times ± SD	2.54 ± 1.31	2.52 ± 1.38	0.94
Total times of bed transfer after KPC-KP isolation, mean times ± SD	1.57 ± 0.95	1.66 ± 0.94	0.72
Antibiotic therapy during the last month			
Piperacillin-tazobactam	6(17.1)	5(17.2)	>0.99
Third-generation cephalosporins	21(60.0)	21(72.4)	0.69
Carbapenems	21(60.0)	13(44.8)	0.53
Quinolones	10(28.6)	3(10.3)	0.22
Fosfomycin	2(5.7)	1(3.4)	>0.99
Aminoglycosides	0	1(3.4)	--
Minocycline	1(2.9)	0	--
Crude mortality	8(22.9)	8(27.6)	0.77
Attributable mortality	6(19.4)^a^	5(17.9)	0.88

Footnotes. Data are no. (%) of patients. RPKP, *rmtB*-positive KPC-producing *K. pneumoniae*; RNKP, *rmtB*-negative KPC-producing *K. pneumoniae*; APACHE II, Acute Physiology and Chronic Health Evaluation II score; ICU, intensive care unit; SD, standard deviation.

^a^ the denominator was the number of patients with infection.

**Figure 1:**
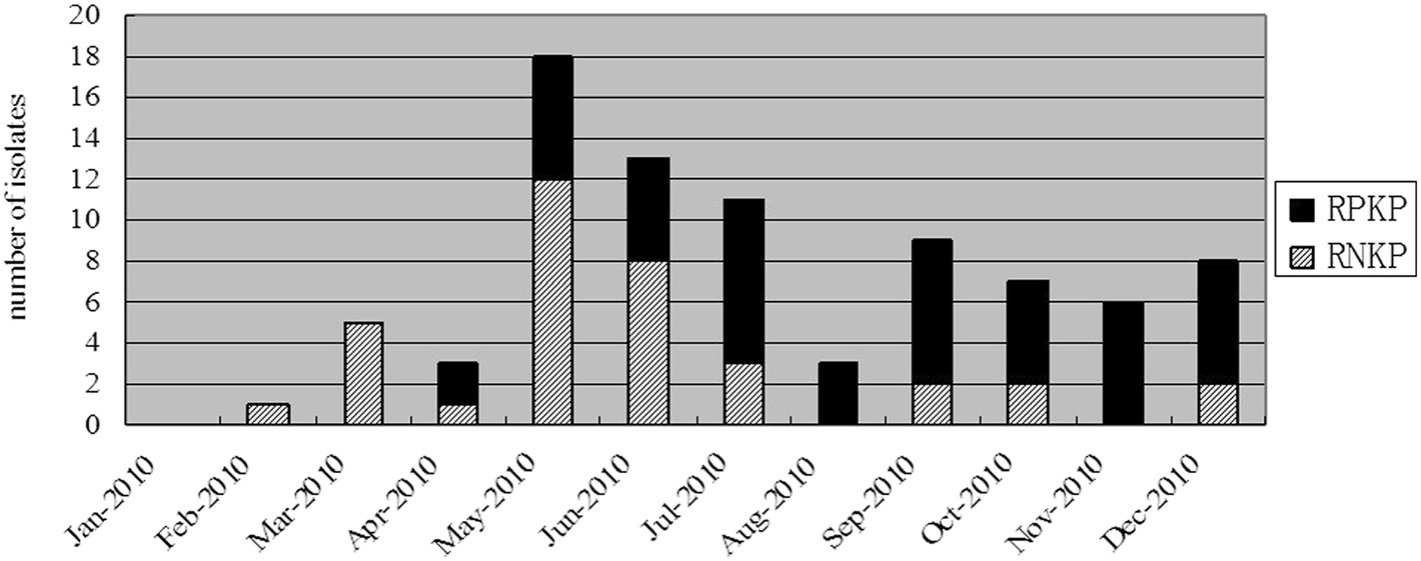
**Temporal distribution of patients infected or colonized with *Klebsiella pneumoniae *carbapenemase 2 (KPC)-producing *K. pneumoniae *during the study period.** RPKP and RNKP are shown separately. RPKP, *rmtB*-positive KPC-producing *K. pneumoniae*; RNKP, *rmtB*-negative KPC-producing *K. pneumoniae.*

The epidemic curve revealed three phases (Figure 1): period 1 (January to April), during which sporadic cases of KPC-KP were identified and RPKP initiated to emerge; period 2 (May to July), during which a hospital-wide outbreak of KPC-KP was observed, with dramatic increase in both RNKP and RPKP isolates, and RPKP became predominant gradually; period 3 (August to December), during which high-level epidemic of KPC-KP persisted, and RPKP overtook RNKP to become the dominant KPC-KP.

KPC-KP isolates were multidrug resistant (Table 2). They only showed sufficient susceptibility to colistin and tigecycline (susceptibility rate: 98.8% for colistin and 96.3% for tigecycline). However, RPKP displayed much severer multidrug resistance phenotypes compared with RNKP. With regard to aminoglycoside antibiotics, all RPKP undoubtedly showed high-level resistance, whereas a majority of RNKP were still susceptible to at least one of them (88.9% for amikacin; 72.2% for netilmicin; 47.2% for gentamicin). In addition, RPKP isolates demonstrated significantly lower susceptibility rates to fosfomycin (8.5% for RPKP versus 88.9% for RNKP, P < 0.01) and minocycline (6.7% for RPKP versus 52.8% for RNKP, P < 0.01).

**Table 2:** Susceptibility profiles of 48 RPKP and 36 RNKP isolates

***Antimicrobial agents***	***MIC range, μg/mL***		***MIC***_***50, ***_***μg/mL***		***MIC***_***90, ***_***μg/mL***		***Susceptible percentage,%***			***P-value***
	**RNKP(n = 48)**	**RPKP(n = 36)**	**RNKP**	**RPKP**	**RNKP**	**RPKP**	**all isolates**	**RNKP**	**RPKP**	
Imipenem	4-256	8-128	128	128	256	128	0	0	0	—
Meropenem	2-256	4-128	64	64	128	64	0	0	0	—
Ertapenem	4-512	8-512	128	128	256	128	0	0	0	—
Cefotaxime	16 to >512	16 to >512	256	>512	512	>512	0	0	0	—
Cefepime	4-256	4-512	128	128	128	512	11.1	11.1	11.1	>0.99
Gentamicin	1 < to >512	256 to >512	8	>512	128	>512	21	47.2	0	<0.01
Netilmicin	1 to >512	256 to >512	8	>512	256	>512	32.1	72.2	0	<0.01
Amikacin	2 to >512	256 to >512	4	>512	>512	>512	39.5	88.9	0	<0.01
Tetracycline	4 to >512	8-256	16	32	512	128	3.7	8.3	0	0.084
Minocycline	2-128	4-32	4	8	64	32	27.2	52.8	6.7	<0.01
Ciprofloxacin	0.25 < to 64	2-64	64	64	64	64	6	13.9	0	0.015
Ofloxacin	0.25 < to 64	1-64	64	64	64	64	8.4	13.9	4.3	0.23
Tigecycline	0.5-4	0.5-4	2	2	2	2	96.3	97.2	95.6	>0.99
Colistin	0.25-256	0.5-2	1	1	2	2	98.8	97.2	100	0.44
Fosfomycin	1 to >1024	2 to > 1024	16	>1024	64	>1024	43.4	88.9	8.5	<0.01

Footnotes. RPKP, *rmtB*-positive KPC-producing *K. pneumoniae*; RNKP, *rmtB*-negative KPC-producing *K. pneumoniae*; MIC, minimum inhibitory concentration; MIC_50_, MIC required to inhibit the growth of 50% of organisms; MIC_90_, MIC required to inhibit the growth of 90% of organisms.

**Figure 2:**
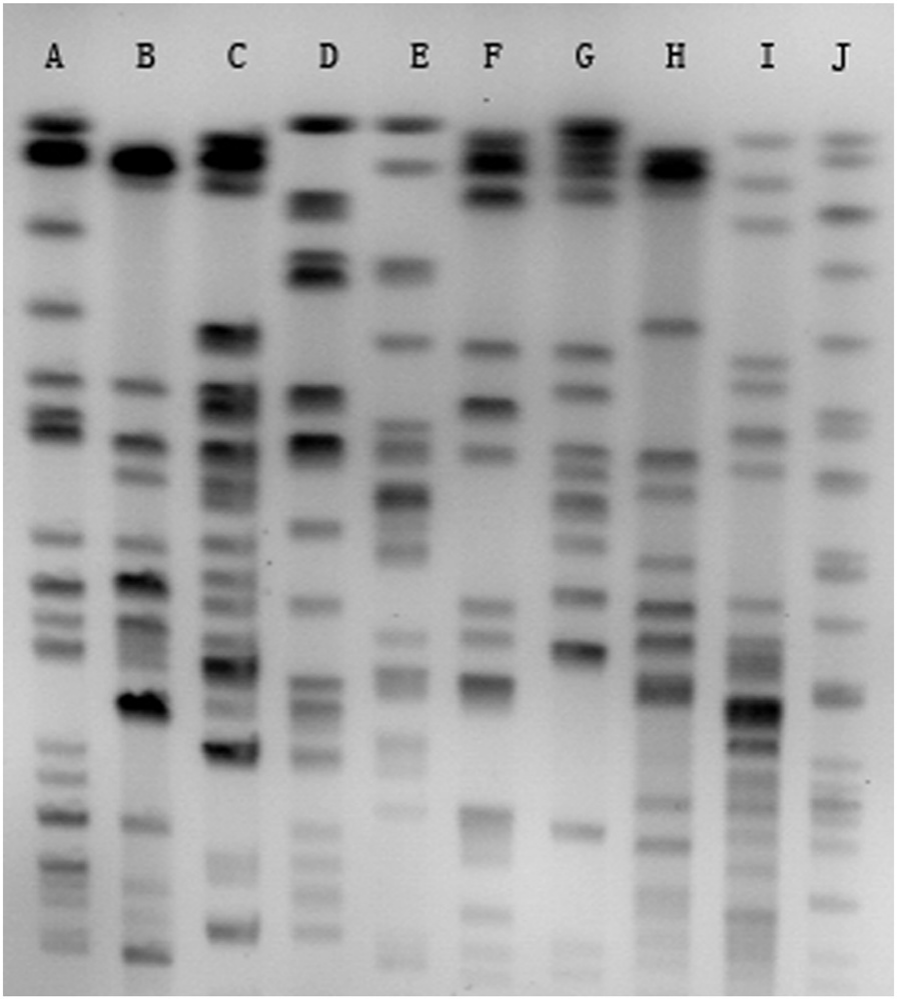
**PFGE patterns of KPC-producing *K. pneumoniae *representing all pulsetypes of KPC-KP isolates.** Lanes A to J, pulsetypes A to J.

Molecular typing results and resistance determinant profiles are shown in Table 3. PFGE analysis revealed 10 different pulsetypes (PTs), designated as A-J (Figure 2). The most widely disseminated PT was A, consisting of 63 clinical isolates (75%). By MLST, 10 distinct sequence types (STs) were identified, including 3 novel STs (ST689, ST690, and ST691). ST11 was the predominant ST, with 68 isolates (80.9%). Most of MLST data were consistent with results generated by PFGE. ST11-PTA was the dominant molecular type in both RPKP and RNKP. However, a greater diversity of STs and more complicated relatedness between the results of PFGE and MLST were observed in RNKP, indicating more diverse genetic backgrounds in RNKP.

**Table 3:** STs, PFGE pulsetypes, and distribution of resistance determinants in 48 RPKP and 36 RNKP isolates

***Groups***	***MLST***	***PFGE***	***Isolate***	***Resistance determinants***
RPKP (n = 48)	11	A	39	*bla*_CTX-M-14_*-bla*_KPC_-*rmtB*-*bla*_SHV_-*bla*_TEM_
	11	B1	4	*bla*_CTX-M-14_*-bla*_KPC_-*rmtB*-*bla*_SHV_-*bla*_TEM_
	11	E	1	*bla*_CTX-M-14_*-bla*_KPC_-*rmtB*-*bla*_SHV_-*bla*_TEM_
	11	I	1	*bla*_CTX-M-14_*-bla*_KPC_-*rmtB*-*bla*_SHV_-*bla*_TEM_
	542	D	1	*bla*_CTX-M-3_*-bla*_KPC_-*rmtB*-*bla*_TEM_
	655	C	2	*bla*_KPC_-*rmtB*-*bla*_SHV_-*bla*_TEM_ (one strain encoded *qnrS*)
RNKP (n = 36)	11	A	22	*bla*_CTX-M-14_*-bla*_KPC_-*bla*_SHV_-*bla*_TEM_ (two strains encoded *qnrS*)
	11	H1	1	*bla*_CTX-M-14_*-bla*_KPC_-*bla*_SHV_-*bla*_TEM_
	214	A	1	*bla*_CTX-M-14_*-bla*_KPC_-*bla*_SHV_
	412	A	1	*bla*_CTX-M-14_*-bla*_KPC_-*bla*_SHV_-*bla*_TEM_-*bla*_OXA-1_-*bla*_OXA-10_-*aac*(*6*)*-Ib*
	412	J	1	*bla*_CTX-M-14_*-bla*_KPC_-*bla*_SHV_-*bla*_OXA-1_-*bla*_OXA-10_-*aac*(*6*)*-Ib*
	412	H2	1	*bla*_CTX-M-14_*-bla*_KPC_-*bla*_SHV_-*bla*_OXA-1_-*bla*_OXA-10_-*aac*(*6*)*-Ib*
	494	F1	4	*bla*_CTX-M-14_*-bla*_KPC_-*bla*_SHV_-*bla*_TEM_
	494	B2	1	*bla*_KPC_-*bla*_SHV_-*bla*_TEM_
	689	E	1	*bla*_CTX-M-3_*-bla*_KPC_-*bla*_SHV_-*bla*_TEM_*-bla*_OXA-1_
	690	F1	1	*bla*_KPC_-*bla*_SHV_-*bla*_TEM_
	691	F2	1	*bla*_KPC_-*bla*_SHV_-*bla*_TEM_
	161	G	1	*bla*_KPC_-*bla*_SHV_-*bla*_TEM_-*bla*_OXA-1_-*bla*_OXA-10_-*qnrS- aac*(*6*)*-Ib*

Footnotes. RPKP, *rmtB*-positive KPC-producing *K. pneumoniae*; RNKP, *rmtB*-negative KPC-producing *K. pneumoniae*; MLST, multilocus sequence typing; PFGE, pulse field gel electrophoresis; STs, sequence types.

Apart from *bla*_KPC-2_ in all isolates and *rmtB* in RPKP, *bla*_CTX-M-14_, *bla*_SHV-12_ and *bla*_TEM-1_ were revealed in most isolates. In addition, four RNKP belonging to ST412 or ST692 also encoded *bla*_OXA-1_–type and *bla*_OXA-10_–type β-lactamases and *aac(6’)-Ib-cr*, and a single ST689 RNKP encoded *bla*_OXA-1_. Only one RPKP and three RNKP strains carried *qnrS*. No other 16S rRNA methylase-encoding genes were identified.

In total, 210 environmental samples were obtained from the general ICU department. Among the 210 samples, only one was RPKP positive, which was identified from a folder of patient chart from an empty bed with a RPKP patient discharged. The environmental RPKP was assigned to ST11-PTA clone, and carried *bla*_CTX-M-14_, *bla*_SHV-12_ and *bla*_TEM-1_ in addition to *bla*_KPC_ and *rmtB* genes. It also showed similar antibiogram as its clinical counterparts (data not shown).

## Discussion

Nowadays, KPC-KP isolates have become major hospital pathogens, and their worldwide spread makes them a great threat to currently available antibiotic-based treatments. Tigecycline, colistin, and aminoglycosides if active, are agents recommended for severe infections caused by carbapenemase-producing *Enterobacteriaceae*[17]. However, despite of good in vitro activity of colistin and tigecycline against most of these strains, clinical data on their in vivo efficacy are quite limited [17]. Emergence of tigecycline resistance and colistin resistance during therapy with these agents is another issue to be considered [18-20]. A colistin-resistant RNKP isolate was also identified from a patient in our study, even without history of colistin therapy. Fortunately, a high proportion of KPC-KP isolates still showed in vitro susceptibility to aminoglycosides [6,21,22], including most of recently emerging colistin-resistant or tigecycline-resistant KPC-KP strains [18-20]. The colistin-resistant isolate found in our study was susceptible to aminoglycosides as well. In addition, a more recent study on KPC-KP bacteriuria demonstrated that treatments including aminoglycosides achieved a significantly higher rate of microbiologic clearance than did treatments with polymyxin B or tigecycline [23]. These findings together indicated that aminoglycosides may serve as effective antimicrobials in treating KPC-KP infections. Emergence of aminoglycoside-resistance in KPC-KP isolates therefore threatens the viability of this therapeutic option and further limits choices of available active agents.

In the present study, we documented the emergence and rapid spread of *rmtB*-positive KPC-KP (RPKP) in a Chinese university hospital. Due to the presence of *rmtB* gene, RPKP isolates showed high level resistance to almost all clinical available aminoglycosides. In addition, RPKP were usually resistant to many more clinically useful antimicrobials compared with RNKP, including minocycline and fosfomycin, which could serve as salvage remedies alone or in combination for infections due to carbapenem-resistant *K. pneumoniae*[21,24]. The fact that RPKP only exhibited sufficient susceptibility to colistin and tigecycline has led to the dead-end of antimicrobial therapy in our hospital, since both colistin and tigecycline were unavailable in China during the study period.

Our results are concordant with the observation that ST11 was the dominant clone of KPC-KP in Mainland China [4]. ST11 is a single-locus variant of the international hyper-epidemic lineage ST258, and has also been identified in Singapore and more recently in Taiwan [25,26]. It is increasingly recognized that ST258 is the predominant clone of KPC-KP across the whole world and has caused outbreaks in many countries [5,6,22]. Furthermore, ST258 has extended to the recently reported colistin-resistant KPC-KP [18,27]. In the present study, ST11 KPC-KP also seemed to show dissemination advantage over other clones and be good colonizers to capture and accumulate resistance determinants. Of particular concern is that increasing prevalence of RPKP coincides with decreasing prevalence of RNKP in our hospital, which might suggest a bigger dissemination advantage of ST11 RPKP over ST11 RNKP, even in the absence of selective pressure. Another particular concern is the fact that 3 RPKP isolates (all belonged to ST11-PTA) were imported from other local hospitals, suggesting the presence of inter-hospital dissemination of this extremely multi-drug resistant pathogen. This indicates a dangerous possibility that ST11 RPKP clone might spread widely outside our hospital in the future.

The fact that most RPKP isolates belonged to ST11-PTA and were acquired in our hospital indicates the possibility that monoclonal cross transmission is the main mode of spreading. However, the failure to find a common environmental reservoir indicated patient-to-patient transmission as the main mechanism of RPKP spread in our study. Frequent bed transfers of case patients, particularly after isolation of RPKP, combined with lack of adequate preventive measures might have facilitated this process. It is also worth mentioning that the only one environmental RPKP isolate was identified from a decontaminated empty bed, which obliged us to enhance the effectiveness of decontamination procedures in our hospital.

Infections with pathogens resistant to more antibiotics have been reported to be associated with poorer functional status and worse outcomes [28,29]. However, in our study, despite of diverse clinical characteristics, no significant differences were observed between the two cohorts. The similar clinical characteristics between RPKP and RNKP patients were likely multifactorial. Firstly, patients in both cohorts were at poor functional status, with severe underlying diseases and highly vulnerable to the colonization or infection of multidrug resistant pathogens. Secondly, RPKP and RNKP were both extremely drug-resistant pathogens. They showed sufficient susceptibility only to colistin and tigecycline. However, neither of them was readily available in China during our study period. Finally, although most RNKP (88.9%) were susceptible to at least one aminoglycoside in vitro, treatment containing aminoglycoside agents was very limited in our hospital, due to limited data on their in-vivo efficacy on KPC-KP infections and their nephrotoxicity [30]. Only three patients with RNKP were treated with antimicrobial regimens containing aminoglycosides in our study, but all of them had good clinical outcomes (data not shown).

## Conclusions

In summary, we documented the emergence and wide dissemination of *rmtB*-positive KPC-KP in a Chinese university hospital. These isolates showed much severer resistance phenotypes compared with their *rmtB*-negative counterparts and seemed to disseminate more easily, representing a major threat for hospitalized patients. Moreover, patients suffered from RPKP infections were usually severely ill and ended up with rather high attributable mortality. These results, together with the fact that discovery of new antibiotics is reduced in recent years, suggest that prudent and conservative use of available active agents combined with good control practices are mandatory.

## Abbreviations

KPC:*Klebsiella pneumoniae* carbapenemase; KPC-KP: *Klebsiella pneumoniae* carbapenemase-producing *K. pneumoniae*; RPKP: *rmtB*-positive *Klebsiella pneumoniae* carbapenemase-producing *K. pneumoniae*; RNKP: *rmtB*-negative *Klebsiella pneumoniae* carbapenemase-producing *K. pneumoniae*; CLSI: Clinical and Laboratory Standards Institute; PFGE: Pulse field gel electrophoresis; MLST: Multilocus sequence typing; BHI: Brain and heart infusion; PTs: Pulsetypes; STs: Sequence types; MIC: Minimal inhibition concentration; PCR: Polymerase chain reaction; ICU: Intensive care unit.

## Competing interests

All authors have no reported conflicts to disclose.

## Authors’ contributions

JJL was involved in all processes related to study design, collecting samples and reviewing medical records, microbiological and molecular studies, analyses of data, and wrote this paper. ZKS and MD contributed to sample collection and microbiological and molecular studies. SB, FSH, HFM, and ZKJ contributed to this study by sample collection. LJL contributed to this study by reviewing and making comments on all drafts of this paper. JFS reviewed and revised this paper, and gave final approval to submit for publication. All authors have read and approved the final manuscript.

## Pre-publication history

The pre-publication history for this paper can be accessed here:

http://www.biomedcentral.com/1471-2334/12/373/prepub
